# Fluorophore-Conjugated Diphenylalanine Peptides Reveal Self-Sorting in a Multicomponent Supramolecular System

**DOI:** 10.3390/ijms27219661

**Published:** 2026-10-28

**Authors:** Yoav Dan, Omri Shelef, Marina Green Buzhor, Linda J. W. Shimon, Ronit Satchi-Fainaro, Doron Shabat, Lihi Adler-Abramovich

**Affiliations:** 1 Department of Oral Biology, The Goldschleger School of Dental Medicine, Gray Faculty of Medical and Health Sciences, Tel-Aviv University, Tel-Aviv 6997801, Israel; 2 Jan Koum Center for Nanoscience and Nanotechnology, Tel-Aviv University, Tel-Aviv 6997801, Israel; 3 The Center for Physics & Chemistry of Living Systems, Tel-Aviv University, Tel-Aviv 6997801, Israel; 4 School of Chemistry, Raymond and Beverly Sackler Faculty of Exact Sciences, Tel-Aviv University, Tel-Aviv 6997801, Israel; 5 Department of Physiology and Pharmacology, Gray School of Medical Sciences, Gray Faculty of Medical and Health Sciences, Tel Aviv University, Tel-Aviv 6997801, Israel; 6 Department of Chemical Research Support, The Weizmann Institute of Science, Rehovot 7610001, Israel; 7 Sagol School of Neuroscience, Tel Aviv University, Tel-Aviv 6997801, Israel

**Keywords:** diphenylalanine, peptide, self-assembly, multicomponent systems, self-sorting, co-assembly, fluorescent peptides, supramolecular nanostructures

## Abstract

The interplay between distinct molecular building blocks in multicomponent supramolecular systems can lead to co-assembly or self-sorting, yet distinguishing between these modes of organization remains challenging. Here, we developed two intrinsically fluorescent derivatives of the self-assembling dipeptide diphenylalanine (FF): nitrobenzoxadiazole-conjugated FF (NBD-FF) and a coumarin-conjugated tert-butyloxycarbonyl-protected FF derivative (BocFF-DAC). Both derivatives formed fibrillar assemblies while exhibiting spectrally distinct fluorescence, enabling their direct visualization by confocal microscopy. Single-crystal X-ray diffraction of NBD-FF revealed an extended hydrogen-bonded lattice in which the NBD moiety participates directly in intermolecular interactions, including aromatic, hydrophobic, and hydrogen-bonding contacts. This finding demonstrates that the fluorophore acts as an active structural element rather than a passive label. When NBD-FF and BocFF-DAC were assembled together, confocal microscopy revealed predominantly spatially distinct fibrillar populations with limited overlap, consistent with preferential self-sorting rather than uniform co-assembly. These findings establish fluorophore-conjugated short peptides as intrinsically traceable building blocks for investigating multicomponent supramolecular organization, while highlighting that fluorophore conjugation can simultaneously enable visualization and influence the assembly.

## 1. Introduction

Peptide self-assembly has emerged as a versatile bottom-up approach for constructing ordered supramolecular structures stabilized by a network of reversible noncovalent interactions, including hydrogen bonding, as well as aromatic, hydrophobic, electrostatic, and van der Waals interactions [1,2]. Minimal peptide building blocks have been extensively explored for the formation of diverse architectures, including fibers, tubes, sheets, vesicles, and hydrogels, yielding functional materials with applications ranging from biomedicine and sensing to electronics and nanotechnology [1,2]. The resulting supramolecular organization can be modulated through variations in peptide sequence, chemical or terminal modification, and assembly conditions such as concentration, solvent composition, pH, and temperature [2,3]. Among minimal peptide systems, diphenylalanine (FF) has become a widely used model for investigating short-peptide self-assembly [4].

The simple FF scaffold can be readily modified at its termini, thereby tuning intermolecular interactions and the morphology and organization of the resulting supramolecular assemblies [3,5]. These effects are also evident for tert-butyloxycarbonyl-FF (BocFF), where N-terminal Boc protection alters the assembly behavior relative to unprotected FF. FF and BocFF exhibit differences in the oligomeric species involved in their early assembly processes, while BocFF itself can adopt different supramolecular states and molecular packing arrangements depending on the assembly conditions [6,7]. The sensitivity of these closely related building blocks to chemical modification and assembly environment highlights the potential complexity introduced when multiple components are combined within the same supramolecular system.

Although single-component peptide systems can display diverse structural and functional properties, a single molecular building block may not readily provide multiple desired functionalities simultaneously. The structural and functional scope of peptide-based materials can therefore be expanded by combining two or more molecular building blocks to generate multicomponent supramolecular systems [2,8,9]. Incorporation of chemically distinct components increases the accessible structural and conformational space and provides opportunities to integrate complementary properties that may not be readily obtained from the individual components alone [8,9]. This concept has been demonstrated in several peptide-based systems, where co-assembly generated properties distinct from those of the individual components. For example, cooperative co-assembly of a pentafluorinated Fmoc-F with Fmoc-K(Fmoc)-RGD produced a multifunctional hydrogel combining antimicrobial activity with biocompatibility and cell-adhesive properties. This strategy was subsequently extended to a multicomponent alginate–cellulose nanofibril matrix, yielding a printable biohybrid hydrogel that retained antibacterial and cell-interactive functionality while providing enhanced structural and mechanical performance [10,11].

The molecular organization of the individual constituents within such multicomponent systems is, however, considerably more complex than in single-component assemblies. Upon mixing, different building blocks may be incorporated into common supramolecular structures through co-assembly or may preferentially associate with molecules of the same type to form compositionally distinct, self-sorted assemblies [8,12,13,14]. These represent limiting modes of organization, while intermediate levels of mixing and segregation may also occur [9,13]. Within FF-based systems, a particularly relevant example is the co-assembly of FF with its N-terminally protected analog BocFF into hybrid supramolecular nanotubes [15]. Increasing the relative proportion of BocFF altered nanotube dimensions and assembly kinetics, while chemical analysis confirmed the presence of both components within the resulting structures [15].

The organization of the different constituents within multicomponent systems is, however, substantially more difficult to predict than the self-assembly of an individual building block. Whether components favor co-assembly or self-sorting depends on their relative molecular compatibility and on the balance of interactions available within and between the different species. Hydrogen-bonding complementarity, aromatic and electrostatic interactions, steric compatibility, and molecular or supramolecular chirality can all shift this balance [12,13,16,17,18]. Environmental and pathway-dependent parameters, including the solvent environment, ionic strength, pH, thermal history, and assembly kinetics, provide additional control over multicomponent organization [12,13,19]. Modulation of nucleation and seed-formation kinetics, for example, can alter the spatial organization of self-sorted peptide-based networks [19]. Consequently, controlling whether structurally related building blocks mix, segregate, or adopt intermediate arrangements remains a central challenge in the design of multicomponent supramolecular materials [13,16,19].

Determining the resulting mode of organization introduces an additional experimental challenge. Spectroscopy, diffraction, and microscopy techniques can provide valuable information on molecular packing, secondary structure, and assembly morphology; however, establishing how chemically distinct components are distributed within individual supramolecular structures can be difficult, particularly when the components generate morphologically similar assemblies [12,20]. Complementary approaches that provide component-specific information are therefore often required to distinguish co-assembly from self-sorting. Fluorescence-based methods are particularly useful in this context, as spectrally distinguishable reporters enable independent visualization of the spatial distributions of individual components, while techniques such as Förster resonance energy transfer (FRET) can provide additional information about molecular proximity [13,19,20,21].

The work of Hamachi and co-workers demonstrated the value of this approach by using selective fluorescent probes to distinguish different supramolecular fiber populations and investigate their spatial and temporal organization using confocal and super-resolution microscopy [19,20,21,22]. Their systems primarily combined chemically distinct self-assembling components, including peptide- and amphiphile- or lipid-based gelators with interaction modes favoring orthogonal organization, while component-selective probes enabled the corresponding assemblies to be differentiated [19,20,21,22]. Fluorescence-based visualization has subsequently been applied to investigate peptide self-sorting in more complex environments, including living systems [23].

An alternative strategy is to covalently incorporate the fluorophore into the peptide itself, thereby associating the fluorescence signal directly with the molecular building block of interest. Pujals et al. demonstrated this concept using N-terminal Cy3- and Cy5-labeled FF derivatives as fluorescent reporters within FF assemblies, enabling investigation of their structural and dynamic properties using fluorescence and super-resolution microscopy [24]. More broadly, aromatic N-terminal modifications are widely employed to tune the assembly and functionality of short peptides, and fluorescent peptide conjugates have themselves been shown to retain the ability to form ordered supramolecular structures [25,26]. Nitrobenzoxadiazole (NBD) is a small fluorescent dye widely used as a tag for peptides and proteins and has also been incorporated into self-assembling peptide systems as a fluorescent reporter [27,28]. Wang and co-workers developed an NBD-functionalized octapeptide, NBD-FFETIGGY, whose self-assembled nanofibers could be directly visualized by fluorescence microscopy in cells [28]. More recently, Carney et al. employed the distinct NBD-labeled peptide NBD-FF-YP to monitor peptide assemblies in breast cancer cells [29].

Coumarin derivatives have likewise been incorporated into self-assembling short peptides, where their fluorescence provides an optical readout while their aromatic structure can also contribute to intermolecular interactions within the assembly [26,30]. In a coumarin-functionalized dipeptide, for example, the chromophore participated in aromatic interactions while the conjugate retained nanofiber formation [30,31].

Here, we aimed to explore how related, yet chemically distinct, building blocks organize when combined within a multicomponent system. Building on these precedents, we developed two spectrally distinguishable fluorescent building blocks based on the FF motif: NBD-conjugated FF (NBD-FF) and a coumarin-conjugated Boc-protected FF derivative (BocFF-DAC). Although both derivatives contain the FF dipeptide motif, BocFF is itself a chemically modified FF analogue with assembly behavior distinct from that of unprotected FF [6,7]. Thus, NBD-FF and BocFF-DAC differ not only in the attached fluorophore but also in their underlying molecular interactions. We therefore investigated their individual self-assembly as well as their co-organization. Both derivatives formed fibrillar supramolecular structures while providing spectrally distinct fluorescence signals suitable for direct visualization. Single-crystal X-ray diffraction analysis of NBD-FF further revealed that the conjugated NBD moiety participates directly in the intermolecular interaction network and the resulting molecular packing. When NBD-FF and BocFF-DAC were assembled together, their distinct fluorescence enabled the spatial distributions of the two components to be resolved within the same sample, revealing predominantly separate fibrillar populations consistent with preferential self-sorting rather than uniform co-assembly. These findings demonstrate the utility of fluorescent FF-based building blocks for resolving component organization within multicomponent supramolecular systems, while the combination of two spectrally distinct fluorescent derivatives, their direct comparison within the same multicomponent system, and the molecular-level structural characterization of NBD-FF further highlight how both the underlying peptide scaffold and the appended fluorophore can contribute to the interactions that govern supramolecular organization.

## 2. Results and Discussion

### 2.1. Design, Synthesis, and Characterization of Fluorophore-Conjugated Peptides

**Figure 1:**
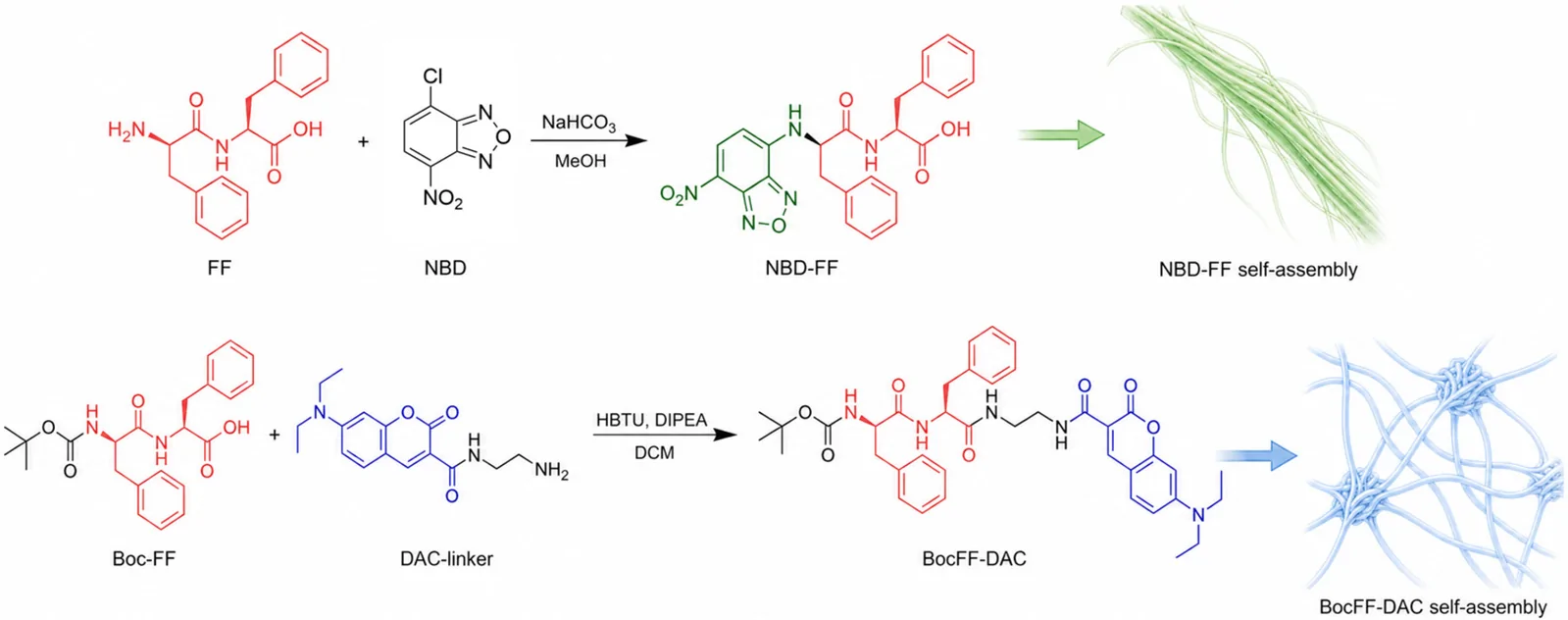
Design, synthesis, and self-assembly of fluorescent FF-based peptide derivatives. NBD-FF was obtained by conjugation of NBD to the free N-terminus of the self-assembling FF peptide. BocFF-DAC was synthesized from the N-terminally Boc-protected FF derivative (BocFF). The coumarin fluorophore was introduced through the C-terminal carboxyl group using an amine-functionalized coumarin linker. Both derivatives contain the FF dipeptide motif but differ in their terminal functionalization and are capable of forming supramolecular fibrillar assemblies (AI-generated via Illustrae).

To enable direct visualization of FF-based supramolecular assemblies, two fluorescent peptide derivatives, NBD-FF and BocFF-DAC, were designed based on the known self-assembling FF and BocFF building blocks, respectively (Figure 1). Both compounds contain the FF core motif; however, BocFF additionally contains an N-terminal Boc group, which increases the molecule’s hydrophobicity and distinguishes its physicochemical and self-assembly behaviors from those of the unprotected FF peptide. Accordingly, different conjugation strategies were employed for the two peptide building blocks. NBD-FF was obtained by direct conjugation of NBD to the free N-terminus of FF (Figures S1–S4). In the case of BocFF, the N-terminus is occupied by the Boc group, and the coumarin fluorophore was therefore introduced through the C-terminal carboxyl group (Figures S5–S11). For this purpose, 7-(diethylamino)coumarin-3-carboxylic acid was first functionalized with an ethylenediamine linker, and the resulting amine-containing coumarin derivative was subsequently coupled to the C-terminus of BocFF to obtain BocFF-DAC. Following purification, the formation of both fluorophore-conjugated peptides was confirmed by high-performance liquid chromatography (HPLC), liquid chromatography–mass spectrometry (LC-MS), and proton nuclear magnetic resonance (^1^H-NMR) analyses, with the observed molecular masses corresponding to the expected products (Figures S2–S4 and S9–S11).

### 2.2. NBD-FF Self-Assembles into Fluorescent Fibrillar Structures

**Figure 2:**
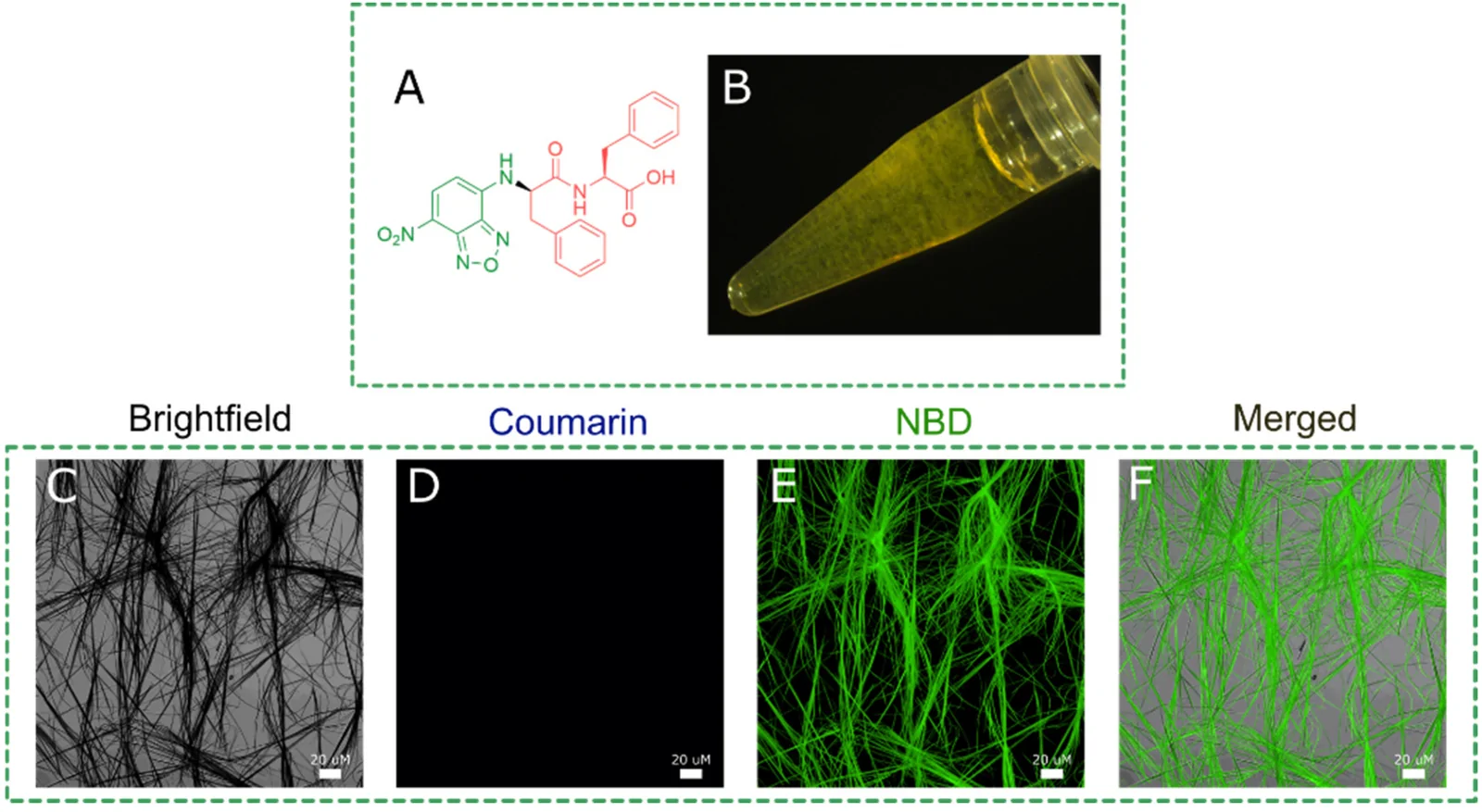
Self-assembly and fluorescence of NBD-FF. (**A**) Molecular structure of NBD-FF, with the NBD moiety highlighted in green. (**B**) Representative image of an NBD-FF sample following solvent-induced self-assembly. (**C**) Brightfield image showing elongated fibrillar NBD-FF assemblies. (**D**) Coumarin fluorescence channel showing negligible signal. (**E**) NBD fluorescence channel showing NBD-FF-containing fibrillar structures. (**F**) Merged brightfield and fluorescence images. Scale bars: 20 µm in (**C**–**F**).

Following successful synthesis and characterization of NBD-FF, we next examined the self-assembly behavior of the NBD-FF conjugate (Figure 2A). NBD-FF assemblies were prepared using a solvent-switch approach by diluting the peptide from 1,1,1,3,3,3-hexafluoro-2-propanol (HFIP) into double-distilled water (DDW). The resulting samples exhibited the characteristic yellow-green appearance associated with the NBD fluorophore (Figure 2B). Brightfield light imaging revealed the formation of an extended network of elongated fibrillar structures, demonstrating that NBD-FF forms elongated fibrillar assemblies (Figure 2C). Confocal fluorescence microscopy further revealed strong NBD fluorescence associated with the fibrillar assemblies, enabling direct visualization of the supramolecular structures (Figure 2C–F). Together, these results demonstrate that NBD-FF forms fibrillar assemblies while simultaneously providing an intrinsic fluorescent signal suitable for visualizing the assembled structures.

### 2.3. Single-Crystal Structure of NBD-FF Reveals Its Intermolecular Interactions

**Figure 3:**
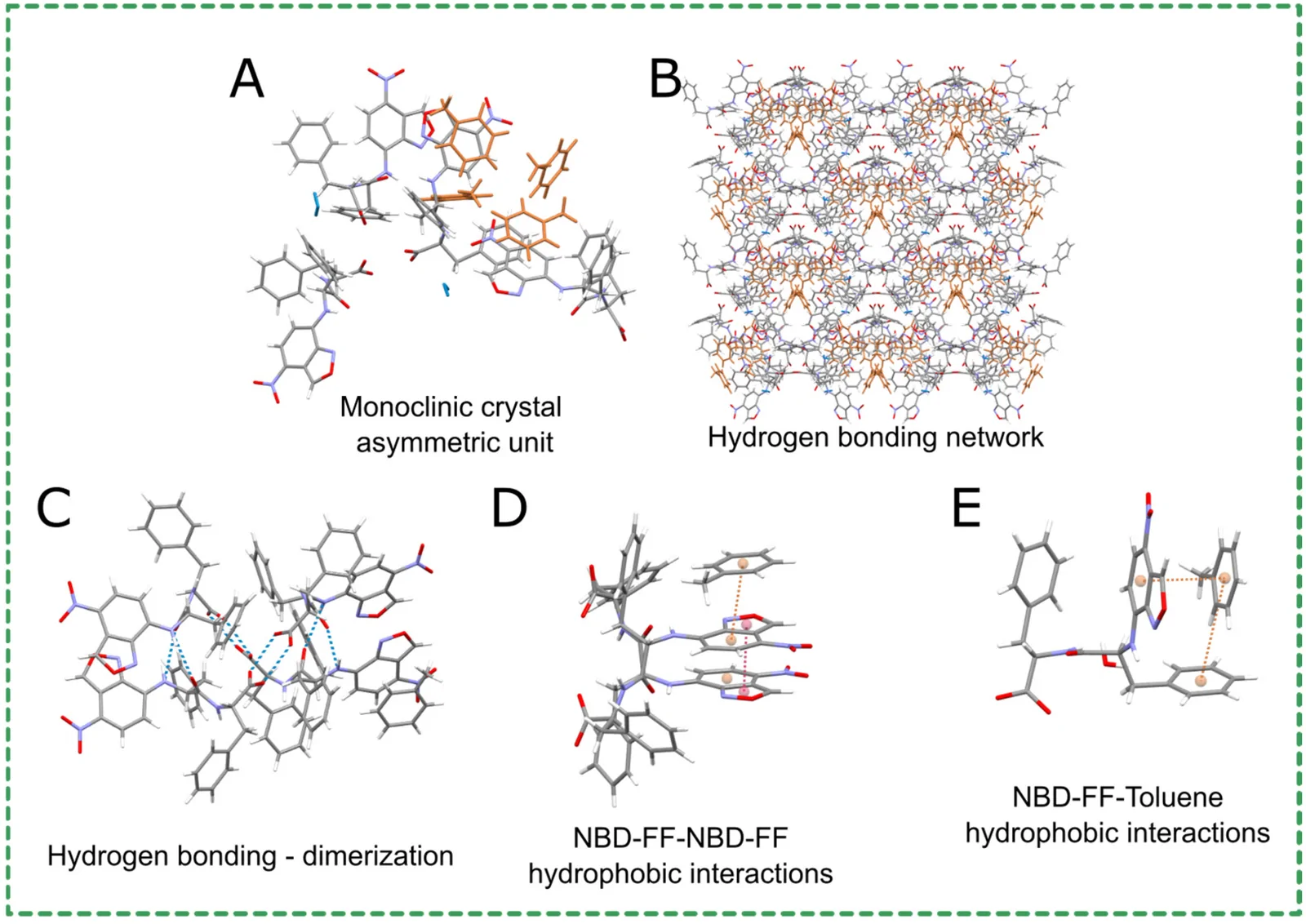
Solvated crystal structure of NBD-FF. (**A**) Asymmetric unit of the monoclinic NBD-FF crystal. (**B**) Crystal packing showing an extended hydrogen-bonded network containing cavities occupied by toluene molecules. (**C**) Intermolecular hydrogen bonding between NBD-FF molecules, including backbone hydrogen bonds and carboxylic acid dimerization. (**D**,**E**) Aromatic and hydrophobic interactions contributing to the crystal packing. (**D**) Face-to-face aromatic stacking between neighboring NBD-FF molecules and between NBD-FF and toluene. (**E**) Additional face-to-face aromatic stacking between NBD and toluene, and an edge-to-face aromatic interaction between a phenylalanine side chain and toluene.

To characterize the molecular packing and noncovalent interactions stabilizing the NBD-FF crystal structure, we set out to grow single crystals for X-ray diffraction (XRD) analysis. Crystals suitable for diffraction were obtained by the slow evaporation of the peptide solution in HFIP/toluene. The aromatic NBD moiety was expected to provide additional sites for aromatic interactions. Furthermore, conjugation of NBD to the N-terminus blocks the free N-terminal amino group and was therefore expected to suppress the conventional head-to-tail interactions commonly associated with free peptide termini. The peptide formed crystals belonging to the monoclinic crystal system, space group I2 (Figure 3A). The asymmetric unit contained four NBD-FF molecules, four toluene molecules, and two additional water molecules. Thus, the obtained crystals represent a solvated structure containing both water and toluene molecules within the crystal lattice. Furthermore, the peptide’s crystal lattice exhibited an extended hydrogen-bonded network (Figure 3B). This network generated solvent-containing cavities within the lattice that were occupied by toluene molecules.

The NBD-FF crystal lattice was stabilized by multiple non-covalent interactions, such as hydrogen bonding, aromatic interactions, and hydrophobic packing (Figure 3C–E). The reported intermolecular interactions represent the overall contacts observed within the asymmetric unit, which contains four crystallographically independent NBD-FF molecules. Hydrogen bonds contributed to lattice stabilization and were observed between peptide N–H donors and carbonyl oxygen acceptors. Hydrogen bonds were observed between the NBD-linked F1 amine N–H group and the F1 carbonyl oxygen of neighboring NBD-FF molecules, with donor–acceptor distances of 2.874–2.954 Å (Figure 3C). The F1 carbonyl oxygen of the same molecule also participated in hydrogen bonding with an F1 amine N–H group of a neighboring molecule, with distances of 2.918–3.016 Å (Figure 3C). The F2 amide N–H group formed a hydrogen bond with one of the oxygen atoms of the NBD nitro group, with a donor–acceptor distance of 3.037 Å. The C-terminus carboxylic acid formed a hydrogen-bonded carboxylic acid dimer with the C-terminal carboxylic acid of a neighboring NBD-FF molecule, with distances of 2.550–2.631 Å (Figure 3C). Additionally, water molecules present in the crystal lattice participated in the hydrogen-bonding network. The water molecules were positioned to form hydrogen bonds with the oxygen atoms of the NBD nitro group, with donor–acceptor distances ranging from 2.881 to 2.948 Å. The water molecules also formed hydrogen-bonding contacts with the F2 amide N–H groups of selected NBD-FF molecules, with distances of 2.824–2.912 Å.

Typically, in FF derivatives, the phenylalanine side chains can participate in aromatic interactions that contribute to stabilization of the supramolecular structure. However, in the NBD-FF crystal, no direct stacking between the phenylalanine aromatic rings was observed. In contrast, prominent aromatic interactions involved the fused heteroaromatic ring system of the NBD moiety. For the aromatic interactions described below, distances are reported as centroid–centroid distances, while the relative orientation of the aromatic rings was used to classify the contacts as face-to-face or edge-to-face. A face-to-face aromatic stacking arrangement was observed between the NBD groups of neighboring NBD-FF molecules, involving their furazan rings, with a distance of 3.319 Å (Figure 3D).

Additional aromatic interactions involved the incorporated toluene molecules. A face-to-face aromatic stacking arrangement was observed between an NBD group and a toluene molecule, with a distance of 3.470 Å (Figure 3D). A further NBD–toluene interaction involved a face-to-face aromatic stacking arrangement between the benzene ring of the NBD moiety and a toluene molecule, with a distance of 3.553 Å (Figure 3E). Furthermore, an aromatic interaction involving a phenylalanine side chain was observed between the F1 phenyl ring and a neighboring toluene molecule, with a geometry consistent with an edge-to-face interaction and a distance of 5.158 Å (Figure 3E).

Analysis of the backbone dihedral angles for the four crystallographically independent molecules within the asymmetric unit revealed highly similar extended backbone conformations. The molecules exhibited consistent φ (phi) dihedral angles ranging from −73.20° to −84.25° for F1 and from −61.39° to −78.89° for F2. Correspondingly, the ψ (psi) dihedral angles ranged from 156.49° to 162.61° for F1 and from 163.08° to 177.77° for F2. These paired (φ,ψ) values are consistent with an extended, PPII-like region of the Ramachandran space, rather than a canonical β-strand conformation. Consistent with this extended geometry, the aromatic phenylalanine side chains were observed to project in opposite directions relative to the peptide backbone. This highly organized conformation may facilitate efficient crystal packing by minimizing steric crowding between adjacent bulky phenyl rings while simultaneously positioning the terminal carboxylic acid groups to favor the observed C-terminal carboxylic acid dimerization motif.

### 2.4. BocFF-DAC Self-Assembles into Fluorescent Fibrillar Structures

**Figure 4:**
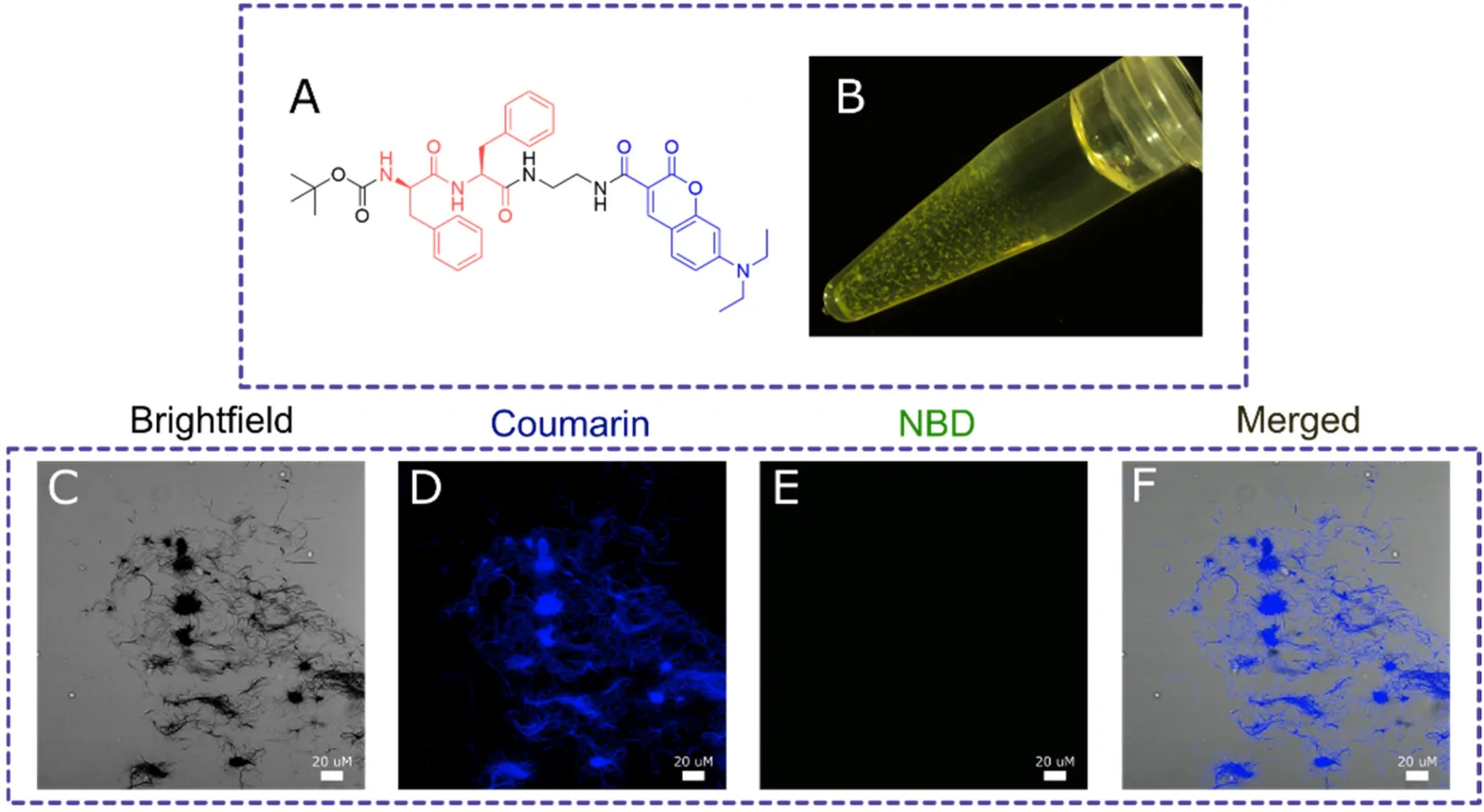
Self-assembly and fluorescence of BocFF-DAC. (**A**) Molecular structure of BocFF-DAC, with the coumarin moiety highlighted in blue. (**B**) Representative image of a BocFF-DAC sample following solvent-induced self-assembly. (**C**) Brightfield image showing fibrillar BocFF-DAC assemblies. (**D**) Coumarin fluorescence channel showing BocFF-DAC-containing fibrillar structures. (**E**) NBD fluorescence channel showing negligible signal. (**F**) Merged brightfield and fluorescence image. Scale bars: 20 µm in (**C**–**F**).

Next, we examined the self-assembly behavior of the BocFF-DAC conjugate (Figure 4). BocFF-DAC assemblies were designed, synthesized and prepared using the same solvent-switch approach by diluting the peptide from HFIP into DDW (Figure 4A,B). Brightfield light imaging revealed the formation of an extensive network of intertwined fibrillar structures, demonstrating that the fluorophore-conjugated peptide forms fibrillar supramolecular assemblies (Figure 4C). Confocal fluorescence microscopy further revealed strong coumarin fluorescence associated with the assembled structures, enabling their direct visualization via the intrinsic fluorescence of the conjugated fluorophore (Figure 4C–F). Together, these observations demonstrate that BocFF-DAC forms fibrillar assemblies while providing a fluorescent signal that enables visualization of the resulting supramolecular assemblies. Attempts to obtain BocFF-DAC single crystals suitable for single-crystal XRD were unsuccessful.

### 2.5. Preferential Self-Sorting of Fluorophore-Conjugated Peptides in a Multicomponent System

**Figure 5:**
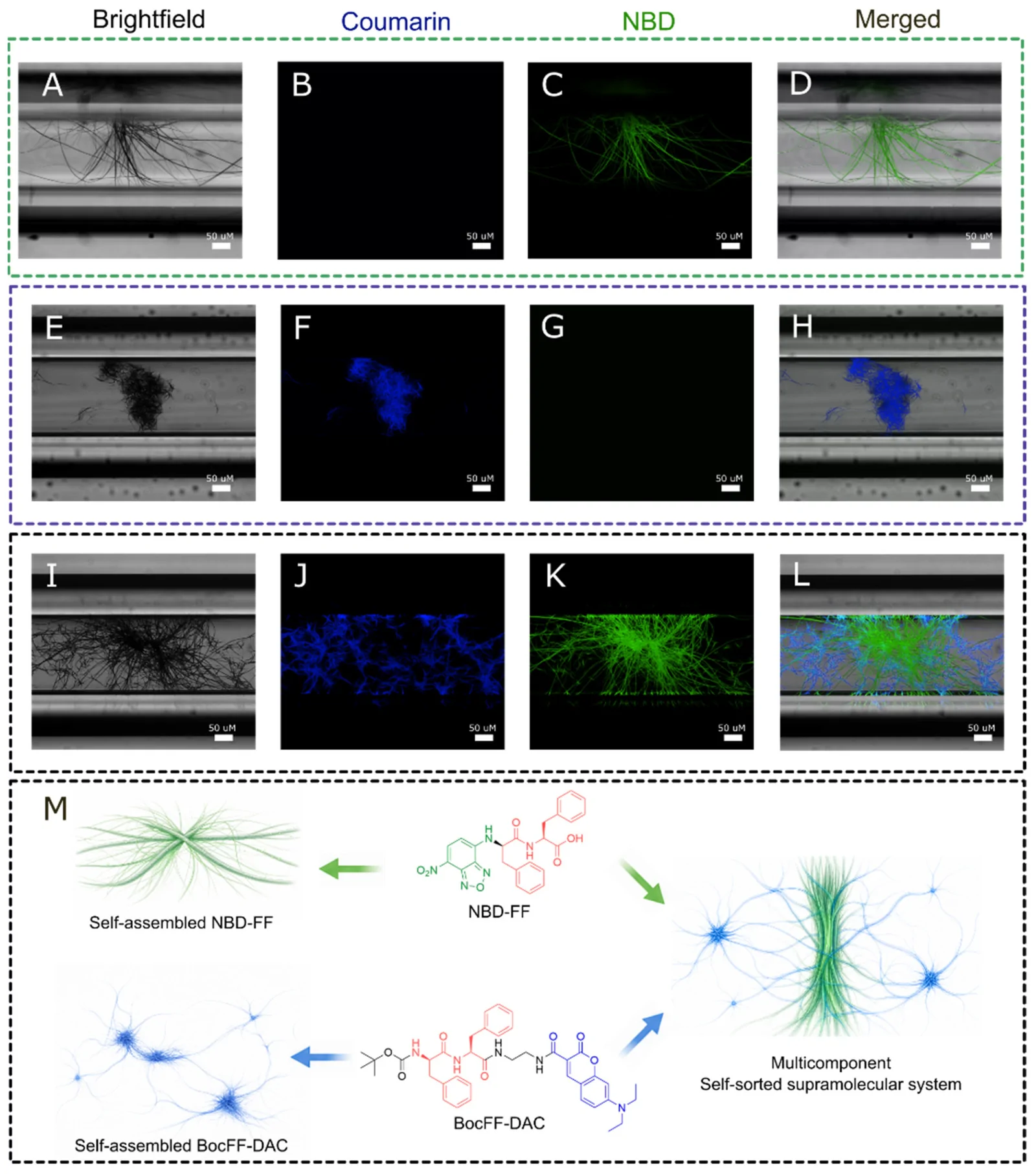
Self-sorting of NBD-FF and BocFF-DAC in a multicomponent supramolecular system. Individual and multicomponent peptide assemblies were imaged within borosilicate glass capillaries using brightfield and confocal fluorescence microscopy. (**A**–**D**) NBD-FF assembled individually. (**A**) Brightfield image showing fibrillar NBD-FF assemblies. (**B**) Coumarin fluorescence channel showing negligible signal. (**C**) NBD fluorescence channel showing NBD-FF-containing fibrillar structures. (**D**) Merged brightfield and NBD fluorescence image. (**E**–**H**) BocFF-DAC assembled individually. (**E**) Brightfield image showing BocFF-DAC assemblies. (**F**) Coumarin fluorescence channel showing BocFF-DAC-containing structures. (**G**) NBD fluorescence channel showing negligible signal. (**H**) Merged brightfield and coumarin fluorescence image. (**I**–**L**) Multicomponent system containing NBD-FF and BocFF-DAC. (**I**) Brightfield image showing the resulting fibrillar assemblies. (**J**) Coumarin fluorescence channel highlighting BocFF-DAC-rich structures. (**K**) NBD fluorescence channel highlighting NBD-FF-rich structures. (**L**) Merged image showing the predominantly spatially distinct localization of BocFF-DAC (blue) and NBD-FF (green), consistent with self-sorting of the two peptide derivatives. (**M**) Schematic representation of the individual self-assembly of NBD-FF and BocFF-DAC and their organization upon combination, illustrating the predominantly spatially distinct populations observed in the multicomponent system (AI-generated via Illustrae). Scale bars: 50 µm in (**A**–**L**).

Having established that both NBD-FF and BocFF-DAC form fluorescent supramolecular assemblies individually, we next investigated their behavior within a multicomponent system. To visualize the assembled structures while minimizing mechanical disturbance during sample handling, the individual peptide assemblies were first reproduced in borosilicate glass capillaries. Samples were loaded into the capillaries following preparation and allowed to assemble prior to imaging, thereby enabling direct observation of the resulting structures in their confined environment (Figure 5A–L).

As controls, NBD-FF and BocFF-DAC were first examined separately under identical imaging conditions, with brightfield, coumarin and NBD fluorescence channels collected (Figure 5A–H). NBD-FF formed elongated fibrillar assemblies that were readily detected in the NBD channel, whereas essentially no corresponding signal was observed in the coumarin channel (Figure 5A–D). Conversely, BocFF-DAC assemblies displayed strong fluorescence in the coumarin channel with negligible signal in the NBD channel (Figure 5E–H). These single-component experiments confirmed that both assemblies could be visualized within the capillaries and, importantly, established the ability to distinguish the two fluorophore-conjugated peptides using their respective fluorescence channels.

We next examined a multicomponent system containing both NBD-FF and BocFF-DAC. The two peptides were combined in HFIP, and assembly was induced simultaneously by switching the solvent to DDW, resulting in final concentrations of 0.5 mM each. Brightfield imaging revealed an extensive population of fibrillar structures within the capillary (Figure 5I). However, separation of the fluorescence channels revealed that these structures were not uniformly composed of both fluorescent peptides. The coumarin channel showed a population of BocFF-DAC-containing fibrillar assemblies distributed throughout the imaged region (Figure 5J), whereas the NBD channel revealed a spatially distinct population of NBD-FF-rich structures (Figure 5K). In the merged image, the blue and green fluorescence signals were predominantly associated with separate fibrillar structures, with limited spatial overlap between the two channels (Figure 5L).

The spatial segregation of the NBD and coumarin fluorescence indicates that, despite being present together during the assembly process, NBD-FF and BocFF-DAC preferentially associate into compositionally distinct supramolecular structures rather than forming uniformly mixed fibrils. This behavior indicates self-sorting of the two fluorophore-conjugated diphenylalanine derivatives within the multicomponent system. Importantly, because the individual peptide controls demonstrate selective detection in their respective fluorescence channels, the distinct blue and green populations observed in the mixed system can be directly attributed to the differential localization of BocFF-DAC and NBD-FF, respectively. Thus, fluorophore conjugation provides not only a means to visualize the individual peptide assemblies, but also a direct approach for resolving their organization and self-sorting behavior within a multicomponent supramolecular system. The resulting organization is characterized by distinct fibrillar populations of NBD-FF and BocFF-DAC rather than a uniformly mixed assembly (Figure 5M).

## 3. Materials and Methods

### 3.1. General Methods

Analytical reverse-phase high-performance liquid chromatography (RP-HPLC) was performed using an UltiMate 3000 Nano LC system (Dionex, ThermoFisher Scientific, Sunnyvale, CA, USA) equipped with a 3000 pump and a VWD-3000 UV–Vis detector, with data acquisition and analysis performed using Chromeleon 6.80 software. A Jupiter C18 reversed-phase column (5 μm particle size, 300 Å pore size, 4.6 × 250 mm) was used. Samples were dissolved in acetonitrile (ACN) and double-distilled water (DDW) containing 0.1% trifluoroacetic acid (TFA) and filtered through a 0.2 μm polyvinylidene fluoride (PVDF) membrane prior to analysis. Chromatographic separation was performed using water containing 0.1% TFA as solvent A and ACN containing 0.1% TFA as solvent B, with a gradient from 30% to 100% solvent B at a flow rate of 1.0 mL min^−1^. The injection volume was 20 μL. Preparative RP-HPLC was performed using an ECOM system equipped with a Phenomenex Luna Axia C18 column (5 μm, 250 × 21.2 mm), using the same mobile-phase system and gradient. Liquid chromatography–mass spectrometry (LC-MS) analysis was performed under the same chromatographic conditions, with mass spectra recorded using a Waters Xevo TQD mass spectrometer. Proton nuclear magnetic resonance (^1^H-NMR) spectra were recorded using a Bruker Avance spectrometer operating at 400 MHz. Chemical shifts are reported in parts per million (ppm) on the δ scale relative to the residual solvent signal (CDCl_3_:δ = 7.26 ppm; DMSO-d_6_:δ = 2.50 ppm).

### 3.2. NBD-FF Synthesis

4-Chloro-7-nitrobenzofurazan (NBD-Cl) (300 mg, 1.5 mmol), FF (516.6 mg, 1.65 mmol, 1.1 equiv.), and NaHCO_3_ (8.9 mmol, 6.0 equiv.) were dissolved in 40 mL methanol (MeOH) and stirred at 55 °C for 3.5 h while monitored using thin-layer chromatography (TLC)/HPLC (Figure S1). The mixture was then diluted with CH_2_Cl_2_ and washed with 1 M HCl. The combined organic layer was then dried over Na_2_SO_4_, and after filtration, the solvent was removed under reduced pressure. Purification was performed via silica gel column chromatography using 1:10 CH_2_Cl_2_/MeOH as an eluent, followed by preparative HPLC to obtain NBD-FF (275 mg, 38.5% yield). The product was then dissolved in ACN and DDW with 0.1% TFA, frozen, and lyophilized to obtain the salt form of NBD-FF. **^1^H NMR** (400 MHz, DMSO) δ 9.31 (s, 1H, -C-N**H**-CH-), 8.72 (s, 1H,-CO-N**H**-CH-), 8.28 (s, 1H, NO_2_-C-C**H**-CH-), 7.34–7.05 (m, 10H, Ar-**H**), 5.97 (s, 1H, NO_2_-C-CH-C**H**-), 4.53 (m, 2H,-NH-C**H**-CO-NH, -NH-C**H**-COOH), 3.13–2.83 (m, 4H, -C**H**_2_-Ar).

### 3.3. 7-(Diethylamino)coumarin-3-carboxylic Acid (Coumarin/DAC) Synthesis

A suspension of 4-(diethylamino)salicylaldehyde (3.86 g, 20 mmol, 1 equiv.) and 2,2-dimethyl-1,3-dioxane-4,6-dione (3.171 g, 22 mmol, 1.1 equiv.) in H_2_O (20 mL) was heated at 110 °C for 3 h while monitored using TLC/HPLC (Figure S5). After cooling, the solid was filtered, washed with ethanol (EtOH), and recrystallized from EtOH to give 7-(diethylamino)coumarin-3-carboxylic acid (2.08 g, 39.9% yield).

### 3.4. Coumarin-NHS (DAC-NHS) Synthesis

DAC (1.0 g, 3.83 mmol, 1 equiv.), N-hydroxysuccinimide (NHS) (0.661 g, 5.7 mmol, 1.5 equiv.) and 1-(3-dimethylaminopropyl)-3-ethylcarbodiimide hydrochloride (EDC·HCl) (0.88 g, 4.6 mmol, 1.2 equiv.) in CH_2_Cl_2_ (50 mL) were stirred at room temperature overnight (Figure S6). The mixture was then diluted with DDW, extracted twice with CH_2_Cl_2_, washed twice with NaHCO_3_ and then with brine. The combined organic layer was then dried over Na_2_SO_4_, and after filtration, the solvent was removed under reduced pressure (1.37 g, 100% yield).

### 3.5. Coumarin-NH-(CH_2_)_2_-NH-Boc (DAC-Linker) Synthesis

DAC-NHS (500 mg, 1.39 mmol, 1 equiv.), N-Boc-ethylenediamine (442 µL, 2.79 mmol, 2 equiv.) and triethylamine (Et_3_N) (729 µL, 4.18 mmol, 3 equiv.) in N,N-dimethylformamide (DMF) (8 mL) were stirred for 20 h at room temperature (Figure S7). The solvent was then removed under reduced pressure, followed by purification performed via silica gel column chromatography using 1/1 to 1/4 hexane (Hex)/ethyl acetate (EtOAc) as an eluent. Solvent was then removed under reduced pressure to obtain DAC-linker (448 mg, 79.6% yield).

### 3.6. BocFF-Coumarin (BocFF-DAC) Synthesis

DAC-linker (418 mg, 1.04 mmol, 1 equiv.) was deprotected using a 1:1 solution of CH_2_Cl_2_:TFA (2 mL) and stirred for 0.5 h at room temperature while monitored by TLC/HPLC. Following deprotection, the solvent and TFA were removed under reduced pressure, and the resulting crude deprotected coumarin-linker was used directly in the subsequent reaction without further purification. The crude deprotected DAC-linker (1.04 mmol, 1 equiv., based on the starting DAC-linker) was then combined with 1.8 mL N,N-diisopropylethylamine (DIPEA) (10.35 mmol, 10 equiv.) and 1 mL CH_2_Cl_2_, and stirred for 0.5 h at room temperature. In parallel, BocFF (470 mg, 1.14 mmol, 1.1 equiv.) was added to 1 mL CH_2_Cl_2_ containing N,N,N′,N′-tetramethyl-O-(1H-benzotriazol-1-yl)uronium hexafluorophosphate (HBTU) (471 mg, 1.24 mmol, 1.2 equiv.) and additional 0.3 mL DIPEA and stirred for 0.5 h at room temperature. The solutions were then combined and stirred for an additional 0.5 h at room temperature, with progress monitored by TLC/HPLC (Figure S8). The mixture was then diluted with CH_2_Cl_2_ and washed using 1 M HCl and brine. The organic layer was then removed under reduced pressure, and purification was performed via silica gel column chromatography using CH_2_Cl_2_/MeOH 5% as the eluent. Solvent was then removed under reduced pressure to obtain BocFF-DAC (470 mg, 65% yield). **^1^H NMR** (400 MHz, CDCl_3_) δ 8.89 (s, 1H, -CH_2_-CH_2_-N**H**-CO), 8.62–8.44 (s, 1H, CO-C-C**H**-C-), 7.37 (dt, *J* = 13.8, 6.1 Hz, 1H, -C-C**H**-CH-), 7.32–6.85 (m, 12H, Ar, CO-N**H**-CH-), 6.71–6.49 (m, 1H, C-CH-C**H**-), 6.50–6.31 (m, 1H, -C-C**H**-C-), 4.53 (m, 1H, -NH-C**H**-CO), 4.30 (s, 1H, -NH-C**H**-CO), 3.57–3.13 (m, 9H, -NH-C**H**_2_-C**H**_2_-NH-, -N-C**H**_2_-CH_3_), 2.97–2.71 (m, 4H), 2.69–2.53 (m, 4H, -C**H**_2_-Ar), 1.30 (s, 9H, Boc), 1.19 (t, *J* = 7.2, 6H, -N-CH_2_-C**H**_3_).

### 3.7. Self-Assembled Sample Preparation

NBD-FF and BocFF-DAC peptide powders were separately dissolved in 1,1,1,3,3,3-hexafluoro-2-propanol (HFIP) to prepare 5 mM stock solutions. Self-assembly was induced by diluting each stock solution with DDW to final peptide concentrations of 0.5 mM and HFIP concentrations of 10% (*v*/*v*).

### 3.8. Multicomponent System Sample Preparation

A mixed peptide stock solution containing 5 mM NBD-FF and 5 mM BocFF-DAC was prepared in HFIP by combining the individual peptide stock solutions. Assembly was induced by diluting the mixed stock solution with DDW to final concentrations of 0.5 mM NBD-FF and 0.5 mM BocFF-DAC, with a final HFIP concentration of 10% (*v*/*v*).

### 3.9. Confocal Microscopy Analysis

Following preparation, peptide samples were either transferred to wells of a black glass-bottom 96-well plate or loaded into borosilicate glass capillaries (0.2 mm inner diameter, 0.1 mm wall thickness). Confocal images were acquired using a ZEISS LSM 900 confocal microscope (ZEISS, Oberkochen, Germany).

### 3.10. Single Crystal X-Ray Diffraction (XRD)

For data collection, crystals were coated in Paratone-N oil (Hampton Research), mounted on a MiTeGen cryo-loop, and flash-frozen in liquid nitrogen. Diffraction data were collected at 100 K at Rigaku Synergy XtaLAB (λ = 1.5418 Å). Data collection and processing were performed using Rigaku CrysAlisPro data stream (version 43). The structures were solved by direct methods using SHELXT and refined by full-matrix least squares against F2 with SHELXL. The crystallographic data file is provided as Supplementary Data S1, and the data are summarized in Supplementary Table S1. The structure has been deposited at the Cambridge Crystallographic Data Centre (CCDC) with deposition number 2588489.

## 4. Conclusions

In this work, two intrinsically fluorescent FF-based building blocks, NBD-FF and BocFF-DAC, were designed and synthesized, and their application for the direct visualization of the organization of short peptides in single-component and multicomponent supramolecular systems was demonstrated. NBD and coumarin were selected as relatively small fluorophores in order to minimize perturbation of the original peptide systems from which they were derived. Nevertheless, despite their relatively small size, the fluorophores are comparable in molecular size and mass to the short FF and BocFF peptide building blocks. Thus, conjugation represents a significant molecular modification that could influence the intermolecular interactions governing peptide assembly.

Notably, NBD-FF and BocFF-DAC were designed from the established self-assembling FF and BocFF scaffolds, respectively. Following fluorophore conjugation, both derivatives were found to form extended fibrillar structures with strong, spectrally distinct fluorescence. Since the parent FF and BocFF systems were not examined under identical conditions in the present study, these observations do not imply preservation of their original morphology or molecular packing. Rather, incorporation of the fluorophores introduced new chemical functionalities and interaction sites that can alter the balance of non-covalent interactions and thereby influence the organization of the resulting assemblies.

This effect was directly demonstrated for NBD-FF using single-crystal XRD. The resulting solvated crystal structure contained water and toluene molecules within cavities formed by an extended hydrogen-bonded network. In addition to interactions involving the peptide backbone and C-terminal carboxylic acid groups, the conjugated NBD moiety participated directly in stabilization of the crystal lattice. The aromatic NBD groups contributed additional hydrophobic and aromatic interactions, while the nitro group participated in hydrogen-bonding interactions. These observations demonstrate that NBD does not function solely as a passive fluorescent label but becomes an integral part of the intermolecular interaction network that governs the resulting supramolecular packing.

The spectrally distinct fluorescence of the two derivatives subsequently enabled their individual organization to be resolved when assembled together. In the multicomponent system, NBD-FF and BocFF-DAC formed predominantly spatially distinct fluorescent fibrillar populations with limited overlap, consistent with preferential self-association and self-sorting rather than uniform co-assembly. Thus, the fluorophores enabled direct visualization of each molecular component, while the NBD-FF crystal structure further demonstrates that fluorophore conjugation can contribute directly to the intermolecular interactions governing molecular packing.

Overall, our findings highlight both the opportunities and considerations associated with using covalently bound fluorophores to investigate short-peptide supramolecular systems. Even relatively small fluorophores can represent substantial modifications when attached to minimal peptide building blocks and may actively participate in interactions determining supramolecular organization. At the same time, their intrinsic and distinct fluorescence provides a powerful approach to directly resolving component distributions and revealing self-sorting in multicomponent peptide assemblies.

## Data Availability

The original contributions presented in this study are included in the article/Supplementary Materials. Further inquiries can be directed to the corresponding author.

## Supplementary Materials

The following supporting information can be downloaded at: https://www.mdpi.com/article/10.3390/ijms27219661/s1.
