# Supplementary material for: Fluorophore-Conjugated Diphenylalanine Peptides Reveal Self-Sorting in a Multicomponent Supramolecular System
Source: Int J Mol Sci. 2026 Oct 28;27(21):9661. doi: 10.3390/ijms27219661 (PMC13763095; doi:10.3390/ijms27219661)

checkCIF/PLATON report

Structure factors have been supplied for datablock(s) lihi18b-sr

THIS REPORT IS FOR GUIDANCE ONLY. IF USED AS PART OF A REVIEW PROCEDURE FOR PUBLICATION, IT SHOULD NOT REPLACE THE EXPERTISE OF AN EXPERIENCED CRYSTALLOGRAPHIC REFEREE.

No syntax errors found. CIF dictionary Interpreting this report

Datablock: lihi18b-sr

Bond precision:	C-C = 0.0113 A		Wavelength=1.54184
Cell:	a=24.3372(2)	b=22.2837(2)	c=25.4168(4)
	alpha=90	beta=111.677(1)	gamma=90
Temperature:	100 K		

	Calculated	Reported
Volume	12809.3(3)	12809.3(3)
Space group	I 2	I 2
Hall group	I 2y	I 2y
Moiety formula	8(C25 H21 N4 O6), 6(C7 H8), 3(H2 O) [+ solvent]	8(C25 H21 N4 O6), 6(C7 H8), 3(H2 O)
Sum formula	C242 H222 N32 O51 [+ solvent]	C242 H222 N32 O51
Mr	4394.52	4394.50
Dx,g cm-3	1.139	1.139
Z	2	2
Mu (mm-1)	0.670	0.670
F000	4612.0	4612.0
F000'	4626.96	
h,k,lmax	30,27,31	29,27,31
Nref	26482[13596]	25856
Tmin,Tmax	0.910,0.935	0.874,1.000
Tmin'	0.910	

Correction method= # Reported T Limits: Tmin=0.874 Tmax=1.000
AbsCorr = MULTI-SCAN

Data completeness= 1.90/0.98 Theta(max)= 75.171

R(reflections)= 0.0873(23028) wR2(reflections)= 0.2629(25856)

S = 1.192 Npar= 1488

The following ALERTS were generated. Each ALERT has the format
test-name_ALERT_alert-type_alert-level.
Click on the hyperlinks for more details of the test.

Alert level B

```
PLAT097_ALERT_2_B Large Reported Max.  (Positive) Residual Density       0.83 eA-3
PLAT230_ALERT_2_B Hirshfeld Test Diff for    C23      --C24      .       14.7 s.u.
PLAT230_ALERT_2_B Hirshfeld Test Diff for    C24      --C25      .        7.9 s.u.
PLAT230_ALERT_2_B Hirshfeld Test Diff for    C39      --C40      .       13.6 s.u.
PLAT230_ALERT_2_B Hirshfeld Test Diff for    C69      --C70      .        9.2 s.u.
PLAT241_ALERT_2_B High   'MainMol' Ueq as Compared to Neighbors of        C24 Check
PLAT241_ALERT_2_B High   'MainMol' Ueq as Compared to Neighbors of        C70 Check
PLAT241_ALERT_2_B High   'MainMol' Ueq as Compared to Neighbors of        C73 Check
PLAT241_ALERT_2_B High   'MainMol' Ueq as Compared to Neighbors of        C88 Check
PLAT242_ALERT_2_B Low    'MainMol' Ueq as Compared to Neighbors of        C72 Check
PLAT340_ALERT_3_B Low Bond Precision on  C-C Bonds ...............    0.01127 Ang.
PLAT411_ALERT_2_B Short Inter H...H Contact  H16      ..H81      .       1.84 Ang.
                                                    x,1+y,z  =      1_565 Check
PLAT417_ALERT_2_B Short Inter D-H..H-D       H8       ..H22A     .       2.04 Ang.
                                                      x,y,z  =      1_555 Check
PLAT420_ALERT_2_B D-H Bond Without Acceptor  O220     --H22B     .     Please Check
PLAT420_ALERT_2_B D-H Bond Without Acceptor  O25      --H25B     .     Please Check
PLAT430_ALERT_2_B Short Inter D...A Contact  O5       ..O12      .       2.63 Ang.
                                         -1/2-x,1/2+y,3/2-z  =      4_456 Check
PLAT430_ALERT_2_B Short Inter D...A Contact  O6       ..O11      .       2.55 Ang.
                                         -1/2-x,1/2+y,3/2-z  =      4_456 Check
PLAT430_ALERT_2_B Short Inter D...A Contact  O17      ..O24      .       2.62 Ang.
                                                      x,y,z  =      1_555 Check
PLAT430_ALERT_2_B Short Inter D...A Contact  O18      ..O23      .       2.63 Ang.
                                                      x,y,z  =      1_555 Check
PLAT990_ALERT_1_B Deprecated .res/.hkl Input Style SQUEEZE Job ...          ! Note
```

Alert level C

```
DIFMX02_ALERT_1_C  The maximum difference density is > 0.1*ZMAX*0.75
            The relevant atom site should be identified.
PLAT084_ALERT_3_C High wR2 Value (i.e. > 0.25) ...................       0.26 Report
PLAT213_ALERT_2_C Atom C39              has ADP max/min Ratio .....        3.3 prolat
PLAT213_ALERT_2_C Atom C70              has ADP max/min Ratio .....        3.1 prolat
PLAT213_ALERT_2_C Atom C73              has ADP max/min Ratio .....        3.4 prolat
PLAT220_ALERT_2_C NonSolvent   Resd 1  C   Ueq(max)/Ueq(min) Range         3.5 Ratio
PLAT220_ALERT_2_C NonSolvent   Resd 2  C   Ueq(max)/Ueq(min) Range         3.6 Ratio
PLAT220_ALERT_2_C NonSolvent   Resd 3  C   Ueq(max)/Ueq(min) Range         5.0 Ratio
PLAT220_ALERT_2_C NonSolvent   Resd 4  C   Ueq(max)/Ueq(min) Range         5.5 Ratio
PLAT222_ALERT_3_C NonSolvent Resd 3   H   Uiso(max)/Uiso(min) Range        4.7 Ratio
PLAT222_ALERT_3_C NonSolvent Resd 4   H   Uiso(max)/Uiso(min) Range        5.0 Ratio
PLAT230_ALERT_2_C Hirshfeld Test Diff for    O3       --C6       .        6.0 s.u.
PLAT234_ALERT_4_C Large Hirshfeld Difference C36      --C37      .       0.20 Ang.
PLAT234_ALERT_4_C Large Hirshfeld Difference C47      --C48      .       0.21 Ang.
PLAT234_ALERT_4_C Large Hirshfeld Difference C63      --C64      .       0.16 Ang.
```

```
PLAT234_ALERT_4_C Large Hirshfeld Difference C64      --C65      .       0.18 Ang.
PLAT234_ALERT_4_C Large Hirshfeld Difference C70      --C75      .       0.18 Ang.
PLAT234_ALERT_4_C Large Hirshfeld Difference C90      --C91      .       0.16 Ang.
PLAT234_ALERT_4_C Large Hirshfeld Difference C200     --C201     .       0.18 Ang.
PLAT234_ALERT_4_C Large Hirshfeld Difference C203     --C204     .       0.22 Ang.
PLAT234_ALERT_4_C Large Hirshfeld Difference C204     --C205     .       0.23 Ang.
PLAT234_ALERT_4_C Large Hirshfeld Difference C205     --C206     .       0.21 Ang.
PLAT241_ALERT_2_C High   'MainMol' Ueq as Compared to Neighbors of        C14 Check
PLAT241_ALERT_2_C High   'MainMol' Ueq as Compared to Neighbors of         O9 Check
PLAT241_ALERT_2_C High   'MainMol' Ueq as Compared to Neighbors of        C38 Check
PLAT241_ALERT_2_C High   'MainMol' Ueq as Compared to Neighbors of        C39 Check
PLAT241_ALERT_2_C High   'MainMol' Ueq as Compared to Neighbors of        C44 Check
PLAT241_ALERT_2_C High   'MainMol' Ueq as Compared to Neighbors of        C47 Check
PLAT241_ALERT_2_C High   'MainMol' Ueq as Compared to Neighbors of        C48 Check
PLAT241_ALERT_2_C High   'MainMol' Ueq as Compared to Neighbors of        C63 Check
PLAT241_ALERT_2_C High   'MainMol' Ueq as Compared to Neighbors of        C89 Check
PLAT242_ALERT_2_C Low    'MainMol' Ueq as Compared to Neighbors of        C20 Check
PLAT242_ALERT_2_C Low    'MainMol' Ueq as Compared to Neighbors of        C23 Check
PLAT242_ALERT_2_C Low    'MainMol' Ueq as Compared to Neighbors of        C36 Check
PLAT242_ALERT_2_C Low    'MainMol' Ueq as Compared to Neighbors of        C40 Check
PLAT242_ALERT_2_C Low    'MainMol' Ueq as Compared to Neighbors of        C43 Check
PLAT242_ALERT_2_C Low    'MainMol' Ueq as Compared to Neighbors of        C45 Check
PLAT242_ALERT_2_C Low    'MainMol' Ueq as Compared to Neighbors of        C69 Check
PLAT242_ALERT_2_C Low    'MainMol' Ueq as Compared to Neighbors of        C71 Check
PLAT242_ALERT_2_C Low    'MainMol' Ueq as Compared to Neighbors of        C75 Check
PLAT242_ALERT_2_C Low    'MainMol' Ueq as Compared to Neighbors of        C86 Check
PLAT242_ALERT_2_C Low    'MainMol' Ueq as Compared to Neighbors of        C90 Check
PLAT242_ALERT_2_C Low    'MainMol' Ueq as Compared to Neighbors of        C91 Check
PLAT243_ALERT_4_C High   'Solvent' Ueq as Compared to Neighbors of       C205 Check
PLAT244_ALERT_4_C Low    'Solvent' Ueq as Compared to Neighbors of       C201 Check
PLAT250_ALERT_2_C Large U3/U1 Ratio for Average U(i,j) Tensor ....        2.1 Note
PLAT250_ALERT_2_C Large U3/U1 Ratio for Average U(i,j) Tensor ....        2.6 Note
PLAT250_ALERT_2_C Large U3/U1 Ratio for Average U(i,j) Tensor ....        3.3 Note
PLAT250_ALERT_2_C Large U3/U1 Ratio for Average U(i,j) Tensor ....        2.9 Note
PLAT250_ALERT_2_C Large U3/U1 Ratio for Average U(i,j) Tensor ....        2.7 Note
PLAT260_ALERT_2_C Large Average Ueq of Residue Including      C200      0.141 Check
PLAT260_ALERT_2_C Large Average Ueq of Residue Including      C300      0.161 Check
PLAT260_ALERT_2_C Large Average Ueq of Residue Including      C400      0.166 Check
PLAT260_ALERT_2_C Large Average Ueq of Residue Including      O220      0.105 Check
PLAT331_ALERT_2_C Small Aver Phenyl C-C Dist C101     --C106     .       1.37 Ang.
PLAT332_ALERT_2_C Large Phenyl C-C Range     C20       -C25      .       0.20 Ang.
PLAT332_ALERT_2_C Large Phenyl C-C Range     C36       -C41      .       0.22 Ang.
PLAT332_ALERT_2_C Large Phenyl C-C Range     C86       -C91      .       0.19 Ang.
PLAT369_ALERT_2_C Long   C(sp2)-C(sp2) Bond  C38      - C39      .       1.53 Ang.
PLAT411_ALERT_2_C Short Inter H...H Contact  H63      ..H63      .       2.13 Ang.
                                                   -x,y,1-z  =      2_556 Check
PLAT411_ALERT_2_C Short Inter H...H Contact  H88      ..H89      .       2.06 Ang.
                                                   -x,y,1-z  =      2_556 Check
PLAT415_ALERT_2_C Short Inter D-H..H-X       H22A     ..H56      .       2.08 Ang.
                                         1/2-x,-1/2+y,3/2-z  =      4_546 Check
PLAT415_ALERT_2_C Short Inter D-H..H-X       H22B     ..H56      .       2.12 Ang.
                                         1/2-x,-1/2+y,3/2-z  =      4_546 Check
PLAT790_ALERT_4_C Centre of Gravity not Within Unit Cell: Resd.  #          1 Note
              C25 H21 N4 O6
PLAT911_ALERT_3_C Missing FCF Refl Between Thmin & STh/L=    0.600         26 Report
PLAT918_ALERT_3_C Reflection(s) with I(obs) much Smaller I(calc) .         46 Check
PLAT975_ALERT_2_C Check Calcd Resid. Dens.  0.44A   From O25             0.89 eA-3
```

```
PLAT977_ALERT_2_C Check Negative Difference Density on H6            -0.31 eA-3
```

Alert level G

```
PLAT003_ALERT_2_G Number of Uiso or Uij Restrained non-H Atoms ...          18 Report
PLAT007_ALERT_5_G Number of Unrefined Donor-H Atoms .............           12 Report
PLAT142_ALERT_4_G s.u. on b - Axis Small or Missing .............      0.00020 Ang.
PLAT187_ALERT_4_G The CIF-Embedded .res File Contains RIGU Records           4 Report
PLAT300_ALERT_4_G Atom Site Occupancy of C300       Constrained at         0.5 Check
PLAT300_ALERT_4_G Atom Site Occupancy of C301       Constrained at         0.5 Check
PLAT300_ALERT_4_G Atom Site Occupancy of C302       Constrained at         0.5 Check
PLAT300_ALERT_4_G Atom Site Occupancy of C303       Constrained at         0.5 Check
PLAT300_ALERT_4_G Atom Site Occupancy of C304       Constrained at         0.5 Check
PLAT300_ALERT_4_G Atom Site Occupancy of C305       Constrained at         0.5 Check
PLAT300_ALERT_4_G Atom Site Occupancy of C306       Constrained at         0.5 Check
PLAT300_ALERT_4_G Atom Site Occupancy of H30A       Constrained at         0.5 Check
PLAT300_ALERT_4_G Atom Site Occupancy of H30B       Constrained at         0.5 Check
PLAT300_ALERT_4_G Atom Site Occupancy of H30C       Constrained at         0.5 Check
PLAT300_ALERT_4_G Atom Site Occupancy of H302       Constrained at         0.5 Check
PLAT300_ALERT_4_G Atom Site Occupancy of H303       Constrained at         0.5 Check
PLAT300_ALERT_4_G Atom Site Occupancy of H304       Constrained at         0.5 Check
PLAT300_ALERT_4_G Atom Site Occupancy of H305       Constrained at         0.5 Check
PLAT300_ALERT_4_G Atom Site Occupancy of H306       Constrained at         0.5 Check
PLAT300_ALERT_4_G Atom Site Occupancy of C400       Constrained at         0.5 Check
PLAT300_ALERT_4_G Atom Site Occupancy of C401       Constrained at         0.5 Check
PLAT300_ALERT_4_G Atom Site Occupancy of C402       Constrained at         0.5 Check
PLAT300_ALERT_4_G Atom Site Occupancy of C403       Constrained at         0.5 Check
PLAT300_ALERT_4_G Atom Site Occupancy of C404       Constrained at         0.5 Check
PLAT300_ALERT_4_G Atom Site Occupancy of C405       Constrained at         0.5 Check
PLAT300_ALERT_4_G Atom Site Occupancy of C406       Constrained at         0.5 Check
PLAT300_ALERT_4_G Atom Site Occupancy of H40A       Constrained at         0.5 Check
PLAT300_ALERT_4_G Atom Site Occupancy of H40B       Constrained at         0.5 Check
PLAT300_ALERT_4_G Atom Site Occupancy of H40C       Constrained at         0.5 Check
PLAT300_ALERT_4_G Atom Site Occupancy of H402       Constrained at         0.5 Check
PLAT300_ALERT_4_G Atom Site Occupancy of H403       Constrained at         0.5 Check
PLAT300_ALERT_4_G Atom Site Occupancy of H404       Constrained at         0.5 Check
PLAT300_ALERT_4_G Atom Site Occupancy of H405       Constrained at         0.5 Check
PLAT300_ALERT_4_G Atom Site Occupancy of H406       Constrained at         0.5 Check
PLAT300_ALERT_4_G Atom Site Occupancy of O25        Constrained at         0.5 Check
PLAT300_ALERT_4_G Atom Site Occupancy of H25A       Constrained at         0.5 Check
PLAT300_ALERT_4_G Atom Site Occupancy of H25B       Constrained at         0.5 Check
PLAT302_ALERT_4_G Anion/Solvent/Minor-Residue Disorder (Resd  7  )        100% Note
PLAT302_ALERT_4_G Anion/Solvent/Minor-Residue Disorder (Resd  8  )        100% Note
PLAT302_ALERT_4_G Anion/Solvent/Minor-Residue Disorder (Resd  9  )        100% Note
PLAT304_ALERT_4_G Non-Integer Number of Atoms in ..... (Resd  7  )        7.50 Check
PLAT304_ALERT_4_G Non-Integer Number of Atoms in ..... (Resd  8  )        7.50 Check
PLAT304_ALERT_4_G Non-Integer Number of Atoms in ..... (Resd  9  )        1.50 Check
PLAT411_ALERT_2_G Short Inter H...H Contact  H38       ..H403      .      2.12 Ang.
                                                    x,y,z  =      1_555 Check
PLAT413_ALERT_2_G Short Inter XH3 .. XHn     H40A      ..H47       .      2.12 Ang.
                                          1/2-x,1/2+y,3/2-z  =      4_556 Check
PLAT413_ALERT_2_G Short Inter XH3 .. XHn     H40B      ..H74       .      1.92 Ang.
                                                    x,y,z  =      1_555 Check
PLAT413_ALERT_2_G Short Inter XH3 .. XHn     H40B      ..H75       .      1.05 Ang.
                                                    x,y,z  =      1_555 Check
PLAT413_ALERT_2_G Short Inter XH3 .. XHn     H40C      ..H75       .      2.14 Ang.
                                                    x,y,z  =      1_555 Check
```

```
PLAT415_ALERT_2_G Short Inter D-H..H-X       H16      ..H25B     .       1.97 Ang.
                                        -1/2-x,1/2+y,3/2-z  =      4_456 Check
PLAT415_ALERT_2_G Short Inter D-H..H-X       H25B     ..H81      .       1.78 Ang.
                                        -1/2-x,1/2+y,3/2-z  =      4_456 Check
PLAT417_ALERT_2_G Short Inter D-H..H-D       H4       ..H25A     .       1.98 Ang.
                                        -1/2-x,1/2+y,3/2-z  =      4_456 Check
PLAT432_ALERT_2_G Short Inter X...Y Contact  O220     ..C31              2.94 Ang.
                                        1/2-x,-1/2+y,3/2-z  =      4_546 Check
PLAT432_ALERT_2_G Short Inter X...Y Contact  C74      ..C400             2.94 Ang.
                                                     x,y,z  =      1_555 Check
PLAT432_ALERT_2_G Short Inter X...Y Contact  C75      ..C400             2.54 Ang.
                                                     x,y,z  =      1_555 Check
PLAT432_ALERT_2_G Short Inter X...Y Contact  C75      ..C401             3.19 Ang.
                                                     x,y,z  =      1_555 Check
PLAT606_ALERT_4_G Solvent Accessible VOID(S) in Structure ........          ! Info
PLAT790_ALERT_4_G Centre of Gravity not Within Unit Cell: Resd.  #          5 Note
              C7 H8
PLAT790_ALERT_4_G Centre of Gravity not Within Unit Cell: Resd.  #          9 Note
              H2 O
PLAT791_ALERT_4_G Model has Chirality at C8          (Sohnke SpGr)          S Verify
PLAT791_ALERT_4_G Model has Chirality at C17         (Sohnke SpGr)          S Verify
PLAT791_ALERT_4_G Model has Chirality at C33         (Sohnke SpGr)          S Verify
PLAT791_ALERT_4_G Model has Chirality at C42         (Sohnke SpGr)          S Verify
PLAT791_ALERT_4_G Model has Chirality at C58         (Sohnke SpGr)          S Verify
PLAT791_ALERT_4_G Model has Chirality at C67         (Sohnke SpGr)          S Verify
PLAT791_ALERT_4_G Model has Chirality at C83         (Sohnke SpGr)          S Verify
PLAT791_ALERT_4_G Model has Chirality at C92         (Sohnke SpGr)          S Verify
PLAT860_ALERT_3_G Number of Least-Squares Restraints .............         85 Note
PLAT869_ALERT_4_G ALERTS Related to the Use of SQUEEZE Suppressed           ! Info
PLAT883_ALERT_1_G No Info/Value for _atom_sites_solution_primary .     Please Do !
PLAT910_ALERT_3_G Missing # of FCF Reflection(s) Below Theta(Min).          4 Note
PLAT912_ALERT_4_G Missing # of FCF Reflections Above STh/L=  0.600        116 Note
PLAT933_ALERT_2_G Number of OMIT Records in Embedded .res File ...         16 Note
PLAT961_ALERT_5_G Dataset Contains no Negative Intensities .......     Please Check
PLAT978_ALERT_2_G Number C-C Bonds with Positive Residual Density.          1 Info
```

---

0 **ALERT level A** = Most likely a serious problem - resolve or explain
20 **ALERT level B** = A potentially serious problem, consider carefully
68 **ALERT level C** = Check. Ensure it is not caused by an omission or oversight
74 **ALERT level G** = General information/check it is not something unexpected

3 ALERT type 1 CIF construction/syntax error, inconsistent or missing data
82 ALERT type 2 Indicator that the structure model may be wrong or deficient
8 ALERT type 3 Indicator that the structure quality may be low
67 ALERT type 4 Improvement, methodology, query or suggestion
2 ALERT type 5 Informative message, check

It is advisable to attempt to resolve as many as possible of the alerts in all categories. Often the minor alerts point to easily fixed oversights, errors and omissions in your CIF or refinement strategy, so attention to these fine details can be worthwhile. In order to resolve some of the more serious problems it may be necessary to carry out additional measurements or structure refinements. However, the purpose of your study may justify the reported deviations and the more serious of these should normally be commented upon in the discussion or experimental section of a paper or in the "special_details" fields of the CIF. checkCIF was carefully designed to identify outliers and unusual parameters, but every test has its limitations and alerts that are not important in a particular case may appear. Conversely, the absence of alerts does not guarantee there are no aspects of the results needing attention. It is up to the individual to critically assess their own results and, if necessary, seek expert advice.

Publication of your CIF in IUCr journals

A basic structural check has been run on your CIF. These basic checks will be run on all CIFs submitted for publication in IUCr journals (*Acta Crystallographica*, *Journal of Applied Crystallography*, *Journal of Synchrotron Radiation*); however, if you intend to submit to *Acta Crystallographica Section C* or *E* or *IUCrData*, you should make sure that full publication checks are run on the final version of your CIF prior to submission.

Publication of your CIF in other journals

Please refer to the *Notes for Authors* of the relevant journal for any special instructions relating to CIF submission.

PLATON version of 13/07/2021; check.def file version of 13/07/2021

Datablock lihi18b-sr - ellipsoid plot

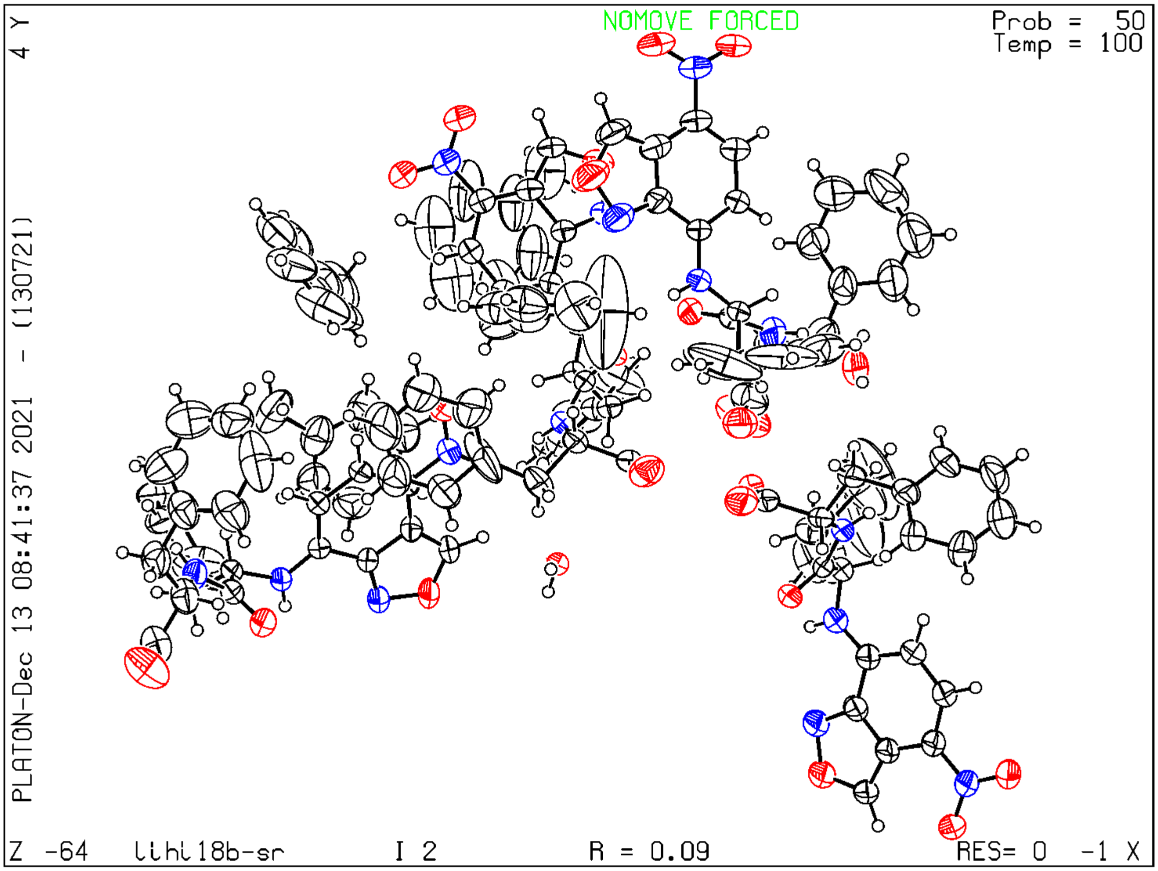

Supplement: Supplementary file 1 [file ijms-27-09661-s001.zip › Supplementary files/Checkcif.pdf]
