# Supplementary material for: Fluorophore-Conjugated Diphenylalanine Peptides Reveal Self-Sorting in a Multicomponent Supramolecular System
Source: Int J Mol Sci. 2026 Oct 28;27(21):9661. doi: 10.3390/ijms27219661 (PMC13763095; doi:10.3390/ijms27219661)
Supplement: Supplementary file 1 [file ijms-27-09661-s001.zip › Supplementary files/SI - Fluorophore-Conjugated Diphenylalanine Peptides Reveal Self-Sorting in a Multicomponent Supramolecular System.pdf]

Supporting information

Fluorophore-Conjugated Diphenylalanine Peptides Reveal Self-Sorting in a Multicomponent Supramolecular System

Yoav Dan[1, 2, 3], Omri Shelef[4], Marina Green Buzhor[5], Linda J. W. Shimon[6], Ronit Satchi Fainaro[5,7], Doron Shabat[4], Lihi Adler-Abramovich[1, 2, 3].*

[1]Department of Oral Biology, The Goldschleger School of Dental Medicine, Gray Faculty of Medical & Health Sciences, Tel-Aviv University, Tel-Aviv 6997801, Israel.
[2]Jan Koum Center for Nanoscience and Nanotechnology, Tel-Aviv University, Tel-Aviv 6997801, Israel.
[3]The Center for Physics & Chemistry of Living Systems, Tel-Aviv University, Tel-Aviv 6997801, Israel.
[4]School of Chemistry, Raymond and Beverly Sackler Faculty of Exact Sciences, Tel-Aviv University, Tel-Aviv 6997801, Israel.
[5]Department of Physiology and Pharmacology, Gray School of Medical Sciences, Gray Faculty of Medical and Health Sciences, Tel Aviv University, Tel-Aviv 6997801, Israel.
[6]Department of Chemical Research Support, The Weizmann Institute of Science, 7610001, Rehovot, Israel.
[7]Sagol School of Neuroscience, Tel Aviv University, Tel-Aviv 6997801, Israel.

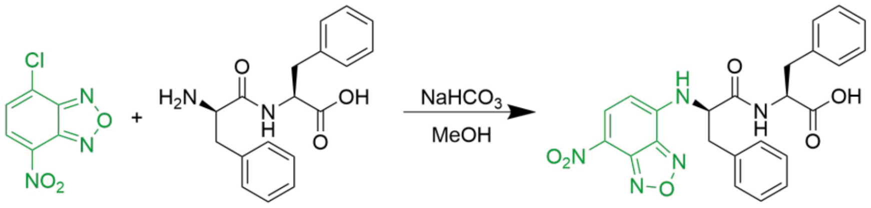

Figure S1. NBD-FF synthesis.

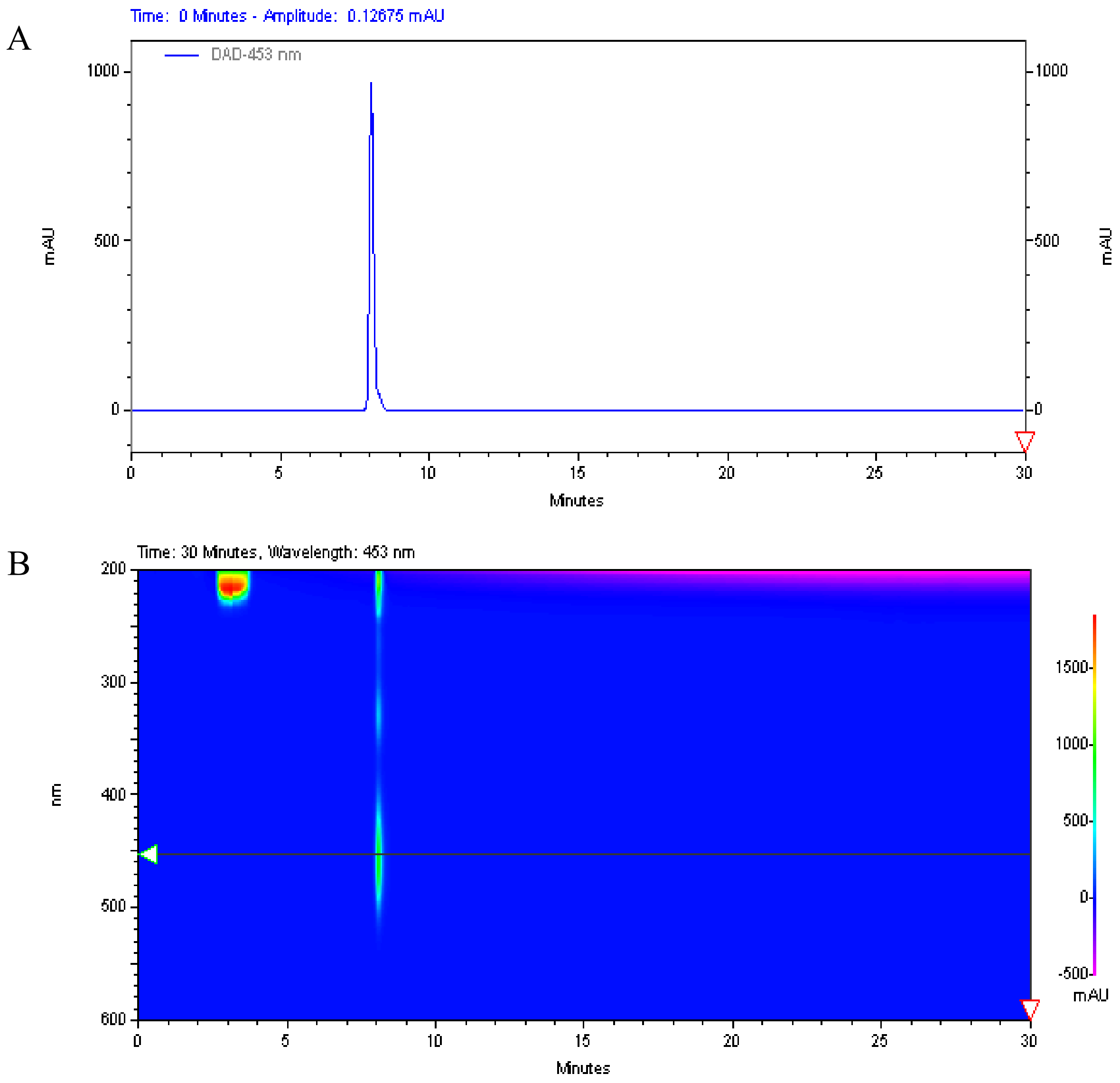

Figure S2. HPLC analysis of NBD-FF. (A) HPLC chromatogram recorded at 453 nm. (B) Spectral plot showing absorbance intensity as a function of retention time and wavelength.

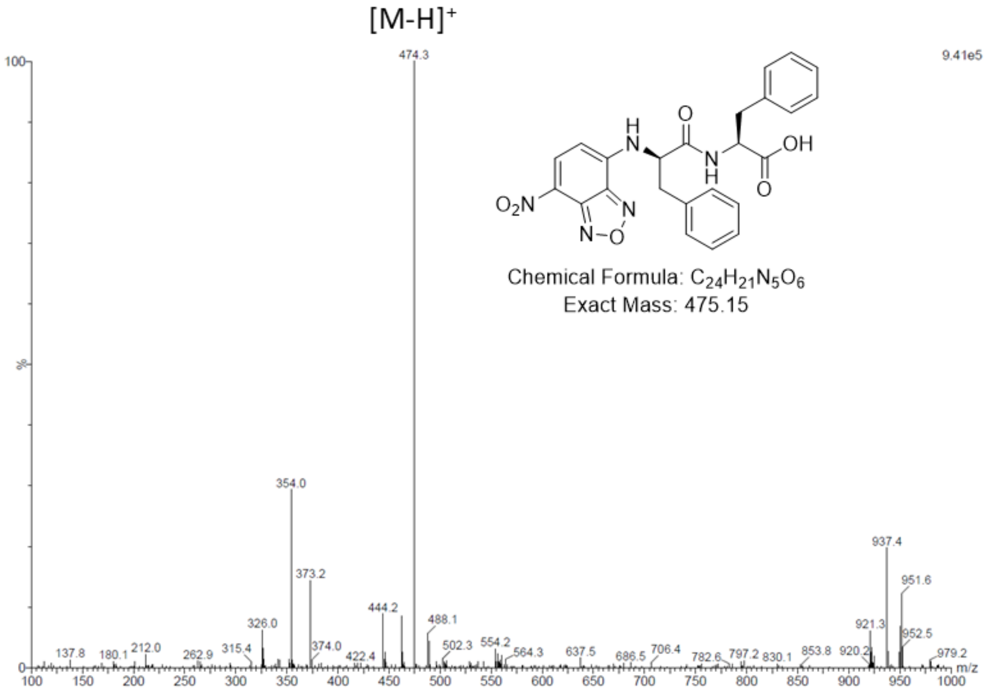

Figure S3. LC-MS analysis of NBD-FF.

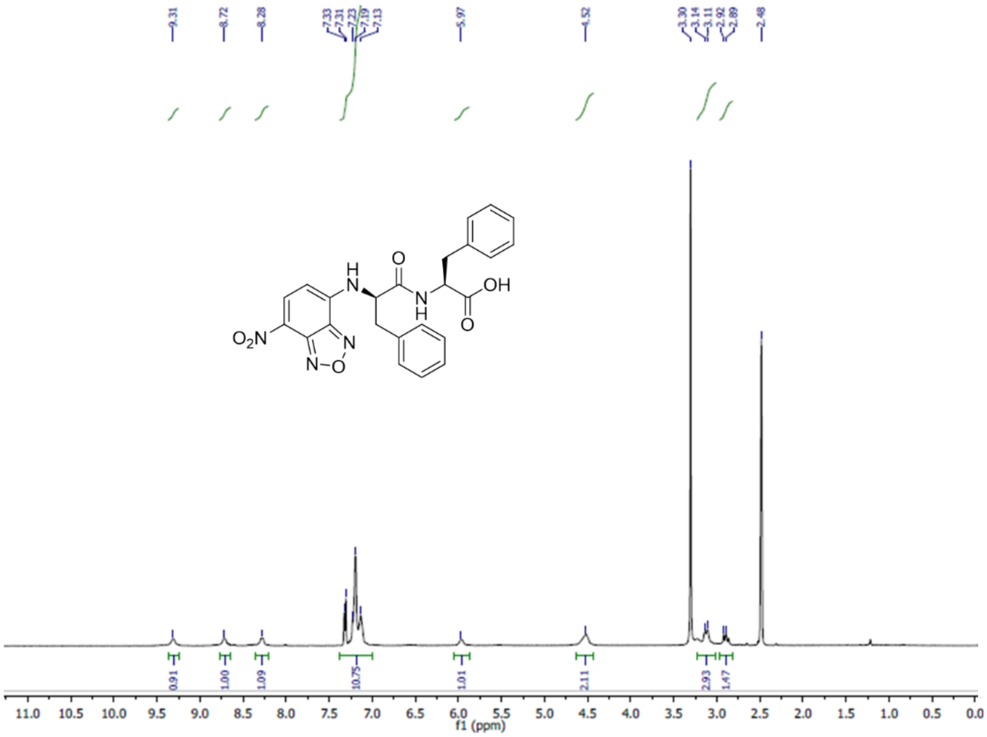

Figure S4. ^{1}H-NMR analysis of NBD-FF in DMSO-d₆.

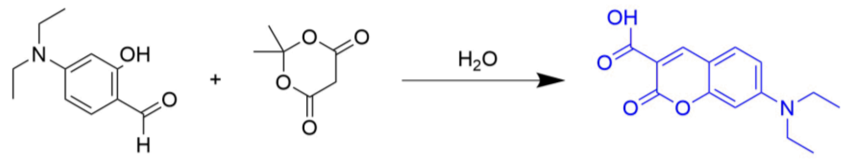

Figure S5. 7-(diethylamino)coumarin-3-carboxylic acid (Coumarin/DAC) synthesis.

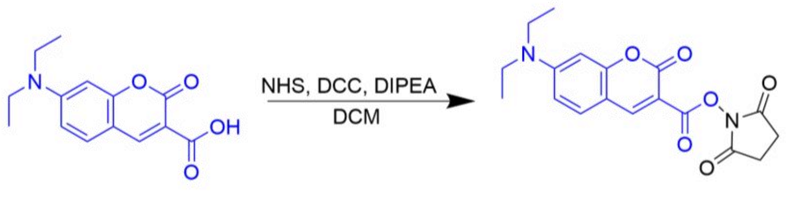

Figure S6. Coumarin-NHS (DAC-NHS) synthesis.

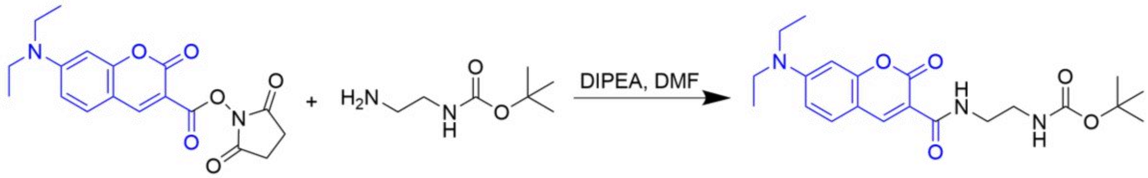

Figure S7. Coumarin-NH-(CH_2)$_2$-NH-Boc (DAC-linker) synthesis.

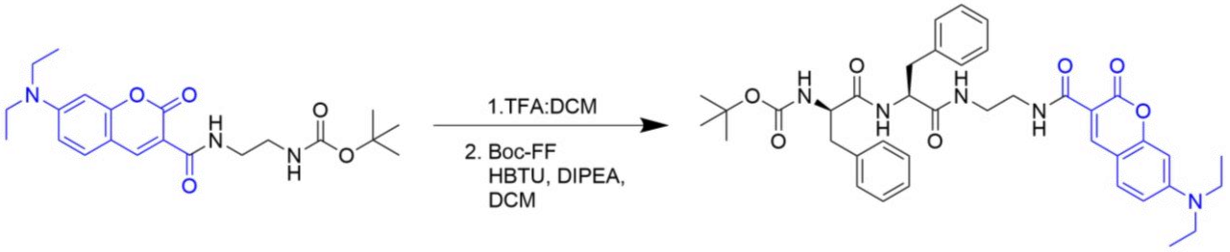

Figure S8. BocFF-Coumarin (BocFF-DAC) synthesis and characterization.

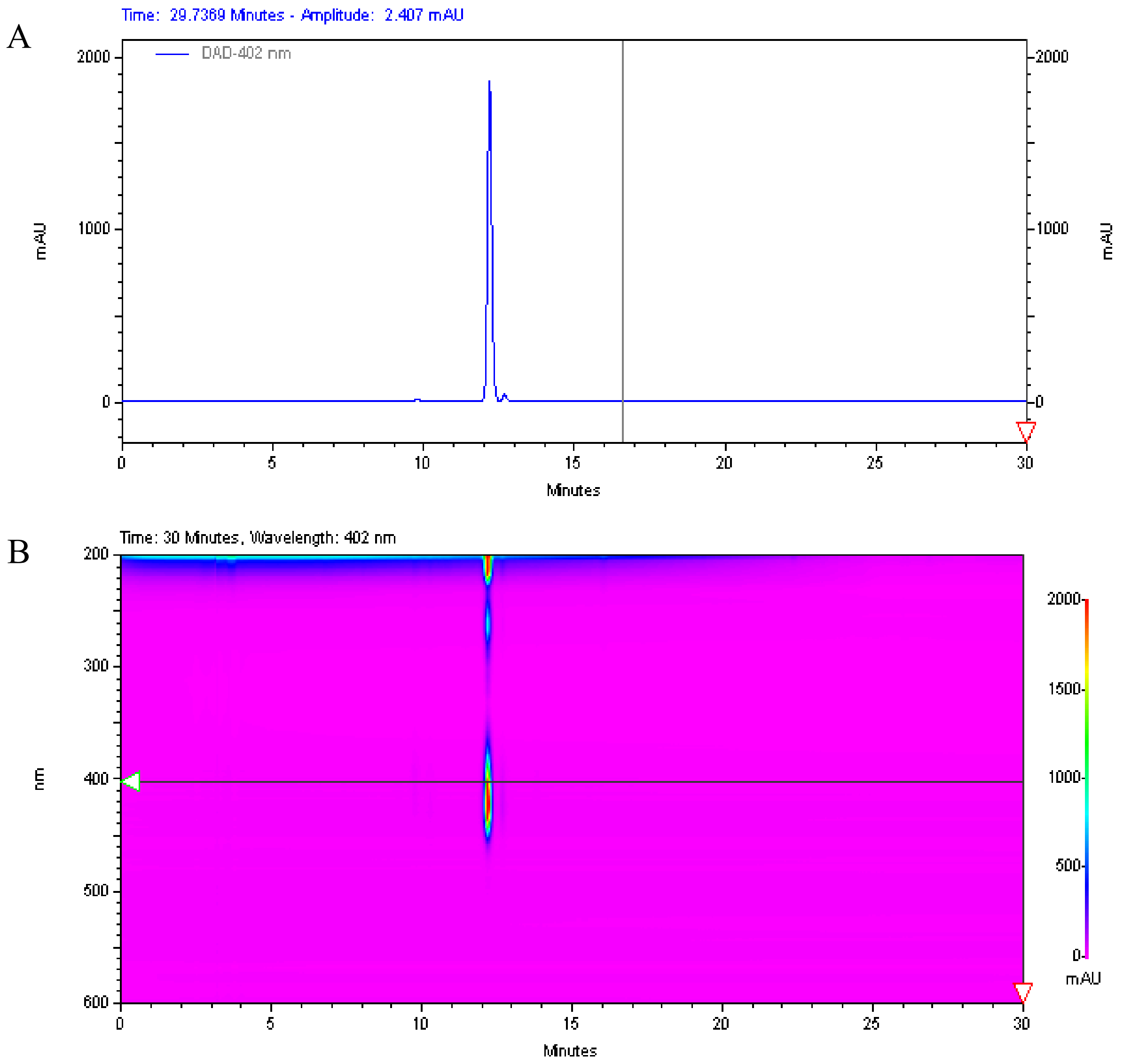

Figure S9. HPLC analysis of BocFF-DAC. (A) HPLC chromatogram recorded at 402 nm. (B) Spectral plot showing absorbance intensity as a function of retention time and wavelength.

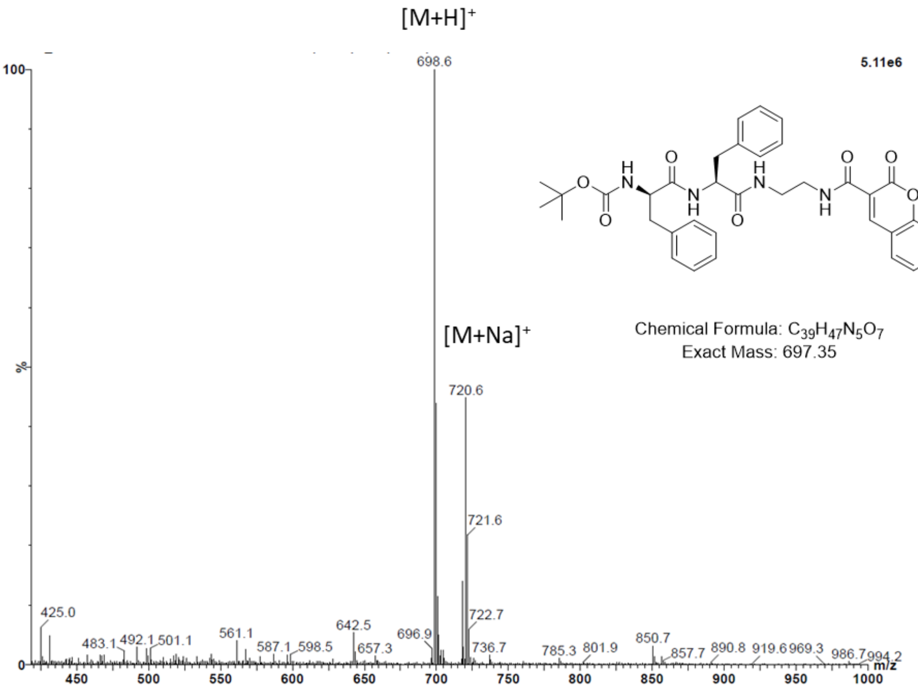

Figure S10. LC-MS analysis of BocFF-DAC.

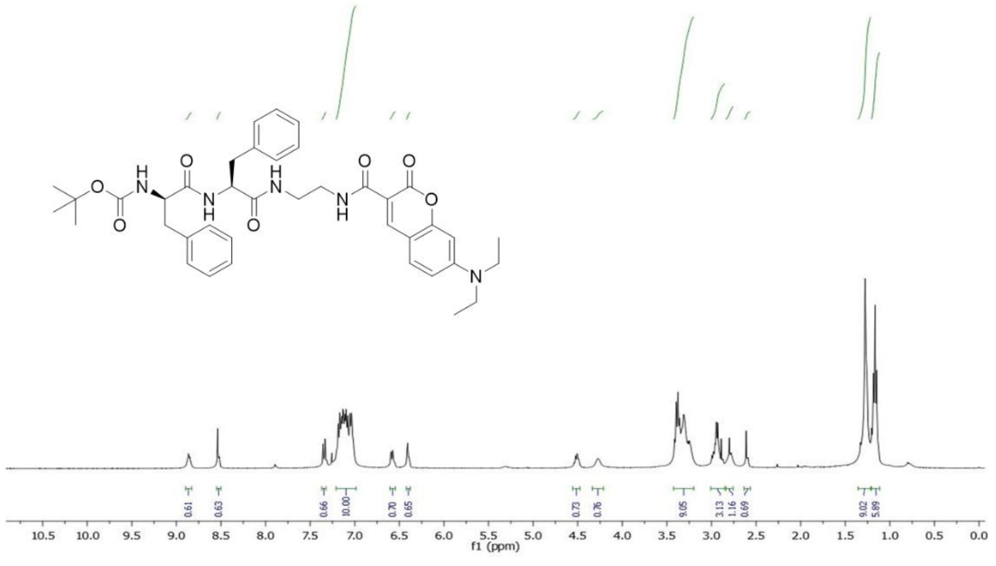

Figure S11. ^{1}H-NMR analysis of BocFF-DAC in $CDCl_3$.

Supplementary Table S1. Crystallographic data of NBD-FF monoclinic single crystal.

**Compound**	**NBDFF Monoclinic**
**CCDC**	2588489
**Crystal description**	Orange needles
**Diffractometer-Source**	Rigaku Synergy XtaLAB
**Empirical Formula**	$C_{242}H_{222}N_{32}O_{51}$
**Formula Weight (g/mol)**	4394.5
**Temperature (K)**	100
**Wavelength (Å)**	1.54184
**Crystal system**	Monoclinic
**Space group**	I2
**a (Å)**	24.3372(2)
**b (Å)**	22.2837(2)
**c (Å)**	25.4168(4)
**α, β, γ, (°)**	90,111.677(1),90
**Volume (Å^3)**	12809.3(3)
**Z**	2
**Density calculated (g/cm^3)**	1.139
**R [I > 2σ(I)]**	0.0873
**No. of reflections [I > 2σ(I)]**	23028
**wR$_2$ (all data)**	0.2629
**No. of reflections (all data)**	25856
